# Ten-Year Surveillance of *Listeria monocytogenes* in Traditional Dry-Cured and Fermented Meat Products of Montenegro

**DOI:** 10.3390/microorganisms14081612

**Published:** 2026-07-23

**Authors:** Amil Orahovac, Ivana Zuber Bogdanović, Marko Nikolić, Aleksandra Martinović, Robert L. Mach

**Affiliations:** 1 Faculty of Food Technology, Food Safety and Ecology, University of Donja Gorica, 81000 Podgorica, Montenegro; ivana.zuber.svl@gmail.com (I.Z.B.); aleksandra.martinovic@udg.edu.me (A.M.); 2 Institute of Chemical, Environmental and Bioscience Engineering, Research Area of Biochemical Technology, TU Wien, 1060 Vienna, Austria; robert.mach@tuwien.ac.at; 3 Diagnostic Veterinary Laboratory, 81000 Podgorica, Montenegro; marco.nikolic.mne@gmail.com

**Keywords:** *Listeria monocytogenes*, traditional meat products, dry-cured ham, fermented sausage, Montenegro, surveillance, food safety, prevalence, Njeguška kobasica, Western Balkans

## Abstract

*Listeria monocytogenes* is a leading cause of foodborne mortality in the European Union, with ready-to-eat (RTE) meat products representing a primary transmission vehicle. Traditional dry-cured and fermented meat products, produced under small-scale artisanal conditions, present particular microbiological challenges, yet systematic surveillance data from the Western Balkans remain limited. Microbiological testing results for *L. monocytogenes* in traditional Montenegrin meat products were extracted from the national food control monitoring system over the period 2016–2025. A total of 2471 samples from 58 product varieties were analysed using EN ISO 11290-1/2 standards. Prevalence was estimated with 95% confidence intervals; differences between product categories were assessed by Pearson chi-square with Bonferroni-corrected pairwise comparisons, and temporal trends by the Cochran–Armitage test. The overall prevalence was 6.6%, ranging from 0.5% (2017) to 14.7% (2024). Dry-fermented sausages (kobasica; 17.2%) and cured belly (pančeta; 9.9%) showed significantly higher prevalence than whole-muscle products (pršut 3.8%; pečenica 1.5%; *p* < 0.001). Njeguška kobasica and its vacuum-packed variant recorded the highest individual prevalence (30.1% and 47.6%, respectively). No significant monotonic trend was detected over the surveillance period (Z = 0.801, *p* = 0.423). Prevalence estimates varied considerably across years without evidence of a monotonic trend, supporting the need for a regulatory shift toward production-environment monitoring and integration of whole-genome surveillance in the traditional meat sector.

## 1. Introduction

*Listeria monocytogenes* is a Gram-positive, facultatively anaerobic foodborne pathogen of global public health significance, responsible for invasive listeriosis—a severe and life-threatening illness characterised by septicaemia, meningitis, meningoencephalitis, and pregnancy-associated complications including miscarriage and neonatal infection [1,2]. Although the incidence of invasive listeriosis remains relatively low compared with other foodborne diseases, its case-fatality rate of approximately 13–16% [2] makes it one of the deadliest foodborne infections under European Union surveillance, and the most lethal among the zoonoses monitored under the One Health approach [3,4]. The European Union One Health 2024 Zoonoses Report documented 3041 confirmed invasive human cases of *L. monocytogenes*, corresponding to a notification rate of 0.69 per 100,000 population, with 301 attributed deaths, and identified listeriosis as the zoonosis with the highest case-fatality rate under EU surveillance; the number of cases reported over the last five years showed a significant increasing trend [4]. This upward trajectory has prompted ongoing regulatory revision at the European level, most recently through Commission Regulation (EU) 2024/2895 [5], which strengthens the food safety criteria for *L. monocytogenes* throughout the entire shelf-life of ready-to-eat (RTE) products.

Human listeriosis is overwhelmingly foodborne, with ready-to-eat products serving as the predominant vehicle of transmission. Among RTE food categories, meat and meat products, particularly fermented, dry-cured, and smoked varieties, have been repeatedly implicated as significant sources of *L. monocytogenes* exposure [6,7,8]. The ability of *L. monocytogenes* to grow at refrigeration temperatures, to tolerate low pH (≥4.0) and elevated salt concentrations (up to ~12% NaCl), and to form persistent biofilms on processing equipment makes it particularly well-adapted to the manufacturing environment of cured meat products [9,10]. Inhibition of growth occurs primarily at water activity values below 0.92 and pH below 4.4, the regulatory thresholds established under Commission Regulation (EC) No 2073/2005 for ready-to-eat foods unable to support the growth of *L. monocytogenes* [11], conditions that are achieved progressively during the dry-curing and fermentation processes but that may not be uniformly attained across all product types or production stages [12,13].

The microbiological safety of traditional dry-fermented and dry-cured meat products presents particular challenges that distinguish this category from industrially manufactured RTE meats. Traditional products are typically produced in small-scale facilities relying on spontaneous fermentation by autochthonous house microbiota, without addition of standardised starter cultures, nitrate/nitrite preservatives at industrial levels, or controlled-atmosphere ripening [14,15]. These manufacturing characteristics, while essential to the sensory identity and cultural value of the products, result in greater variability in physicochemical end-points and reduced control over the microbial succession during fermentation and ripening. Consequently, studies of artisanal fermented sausage production across Europe have reported *L. monocytogenes* prevalence values substantially higher than those observed in industrial RTE meat sectors, ranging from 11.6% in Turkish sucuk [16] to 31.5% in food samples and 68.5% in environmental swabs from Sardinian small-scale producers [17], with similar findings reported across small-scale European fermented sausage facilities [18]. Comparable regional data from the Western Balkans confirm this pattern: a recent food-chain survey in Kosovo (2016–2022) reported an overall *L. monocytogenes* prevalence of 11.76% across 995 samples, with 6.33% positivity in ready-to-eat products [19].

Montenegro possesses a rich tradition of dry-cured and fermented meat production, with several products of substantial cultural, economic, and geographical-indication significance. Njeguški pršut and Njeguška kobasica, produced according to traditional methods in the Njeguši region of the Lovćen mountain, hold formal protected geographical status under the Montenegrin national registry and represent flagship products of the country’s culinary heritage [20]. A broader set of regionally identified products, including Crnogorski pršut, Podlovćenska pršuta, Ćeklićka pančeta, Lovćenska kobasica, and traditional fermented beef products such as goveđi sudžuk and goveđi kulen, share characteristic processing technologies based on dry-salting, smoking with autochthonous wood species, and extended air-drying under ambient mountain conditions [20,21]. These products are produced both by small-scale artisanal manufacturers and by mid-sized industrial facilities operating under variable degrees of process standardization [22,23].

Despite the cultural and economic importance of these products and their inherent microbiological risk profile, comprehensive surveillance data on *L. monocytogenes* prevalence in traditional Montenegrin meat products are scarce in the peer-reviewed literature. Previous molecular characterisation studies have documented the presence of persistent *L. monocytogenes* clones in the Montenegrin food chain, including ST8, ST9, ST121, and ST155, with strong evidence of niche adaptation and clonal persistence within processing environments [22,23]. However, these studies have focused primarily on genotypic characterisation of isolates rather than on systematic estimation of prevalence across the broader product spectrum, and have not addressed temporal trends or product category-specific risk patterns over an extended surveillance period.

The aim of the present study was therefore to provide the first comprehensive, decade-long assessment of *L. monocytogenes* occurrence in traditional Montenegrin dry-cured and fermented meat products, based on data generated within the national food control monitoring system between 2016 and 2025. Specific objectives were to estimate the overall and annual prevalence of *L. monocytogenes* across traditional product categories; to characterise category-specific and product-specific contamination profiles, with particular attention to the geographically and culturally significant varieties; to evaluate whether a statistically significant temporal trend in prevalence has emerged over the surveillance period; and to interpret the observed contamination patterns in the context of the established microbial ecology of *L. monocytogenes* in artisanal meat processing environments. The results are intended to provide a microbiological baseline for traditional Montenegrin meat products, inform risk-based prioritisation of surveillance and control activities, and complement existing molecular characterisation studies of *L. monocytogenes* in the Montenegrin food chain.

## 2. Materials and Methods

### 2.1. Data Source and Study Design

Occurrence data were obtained from the national food control monitoring system of Montenegro and covered the period from 2016 to 2025. The dataset included microbiological testing results for *Listeria monocytogenes* in meat products collected within official control programmes. Official control programmes encompassed the monitoring of microbiological food safety criteria at both the production and marketing stages and were implemented in accordance with applicable national and European food safety legislation. Sampling was performed within risk-based inspection plans defined by competent authorities, and all food products covered by official controls were tested in an authorised laboratory according to EN ISO 11290-1 (qualitative detection) and EN ISO 11290-2 (enumeration) standards [24,25].

For each record, the dataset contained information on the year of sampling, product name, type of inspection, number of analysed samples, and the reported analytical result. Results were expressed as either qualitative (not detected; detected) or, where applicable, quantitative (CFU/g). The acceptability of tested products or batches was assessed in relation to the absence, presence, or enumerated count of the organism per unit mass, as defined by Commission Regulation (EC) No. 2073/2005 [11].

### 2.2. Definition and Selection of Traditional Meat Products

From the complete national dataset, a subset of records corresponding to traditional Montenegrin meat products was extracted for the purposes of this study. Traditional meat products were defined operationally as dry-cured, dry-salted, and/or smoked meat products manufactured according to traditional processing technologies characteristic of the Montenegrin region, irrespective of formal geographical indication (GI) status. This definition encompassed products bearing regional designations (e.g., Njeguška, Crnogorska, Podlovćenska, Lovćenska, Ćeklićka) as well as regionally produced fermented beef products (sudžuk, kulen) that are integral to the traditional meat processing heritage of Montenegro.

A total of 58 product varieties meeting the inclusion criteria were identified and retained for analysis, distributed across seven product categories: prosciutto (pršut), dry-fermented sausage (kobasica), cured belly (pančeta), fermented beef sausage (sudžuk), spiced fermented sausage (kulen), cured loin (pečenica), and cured neck (vrat). Products classified as cooked, emulsified, or of non-Montenegrin traditional origin were excluded. Records with fewer than one analysable sample or missing product category information were also removed prior to analysis.

### 2.3. Prevalence Estimation

For each product category and year, the number of *L. monocytogenes*-positive samples and the total number of samples tested were aggregated. A sample was classified as positive if the analytical result indicated detection, irrespective of quantitative concentration. Point prevalence estimates and 95% confidence intervals were calculated using the exact binomial (Clopper–Pearson) method, which provides conservative and accurate interval coverage across a wide range of proportions and sample sizes, including near-boundary values. Prevalence was expressed as the percentage of samples in which *L. monocytogenes* was detected relative to the total number of samples tested within each category and year.

### 2.4. Statistical Analysis

Differences in *L. monocytogenes* prevalence across product categories were assessed using the Pearson chi-square test of independence. Post hoc pairwise comparisons between all category pairs were performed using pairwise chi-square tests with Bonferroni correction for multiple comparisons; adjusted *p*-values below 0.05 were considered statistically significant. The presence of a linear temporal trend in annual prevalence was evaluated using the Cochran–Armitage trend test, with a two-sided significance threshold of *p* < 0.05. All statistical analyses were performed in R (version 4.5.1) using the base stats package and the *DescTools* package (version 0.99.60) for trend analysis [26].

### 2.5. Data Availability

The dataset supporting the findings of this study was obtained from the national food control monitoring system of Montenegro and is available from the corresponding author upon reasonable request, subject to institutional approval.

### 2.6. Ethical Approval

This study was conducted using data obtained from routine official food control monitoring programmes. No interventionary procedures involving animals or humans were performed, and accordingly, ethical approval was not required.

## 3. Results

### 3.1. Overview of the Dataset

A total of 2471 samples belonging to 58 distinct traditional Montenegrin meat product varieties were examined for the presence of *Listeria monocytogenes* over a ten-year surveillance period (2016–2025). Five additional samples originating from third-party accredited laboratories (targeted sampling based on prior suspicion of contamination; 5/5 positive) were excluded from the statistical analyses because their non-random nature would bias prevalence estimates and are described separately in Section 3.6. Samples were distributed across seven product categories: prosciutto (pršut; *n* = 889), dry-fermented sausage (kobasica; *n* = 406), cured loin (pečenica; *n* = 266), cured belly (pančeta; *n* = 252), fermented beef sausage (sudžuk; n = 207), cured neck (vrat; *n* = 242), and spiced fermented sausage (kulen; *n* = 209). The majority of samples originated from domestic production facilities tested under contractual monitoring (*n* = 1116; 45.2%) or on-request inspection (*n* = 617; 25.0%), with smaller proportions subjected to official veterinary inspection monitoring of production (*n* = 105; 4.2%) and retail (*n* = 234; 9.5%). Imported products accounted for 85 samples (3.4%), all of which tested negative throughout the entire surveillance period.

### 3.2. Overall Prevalence and Annual Trends

**Figure 1:**
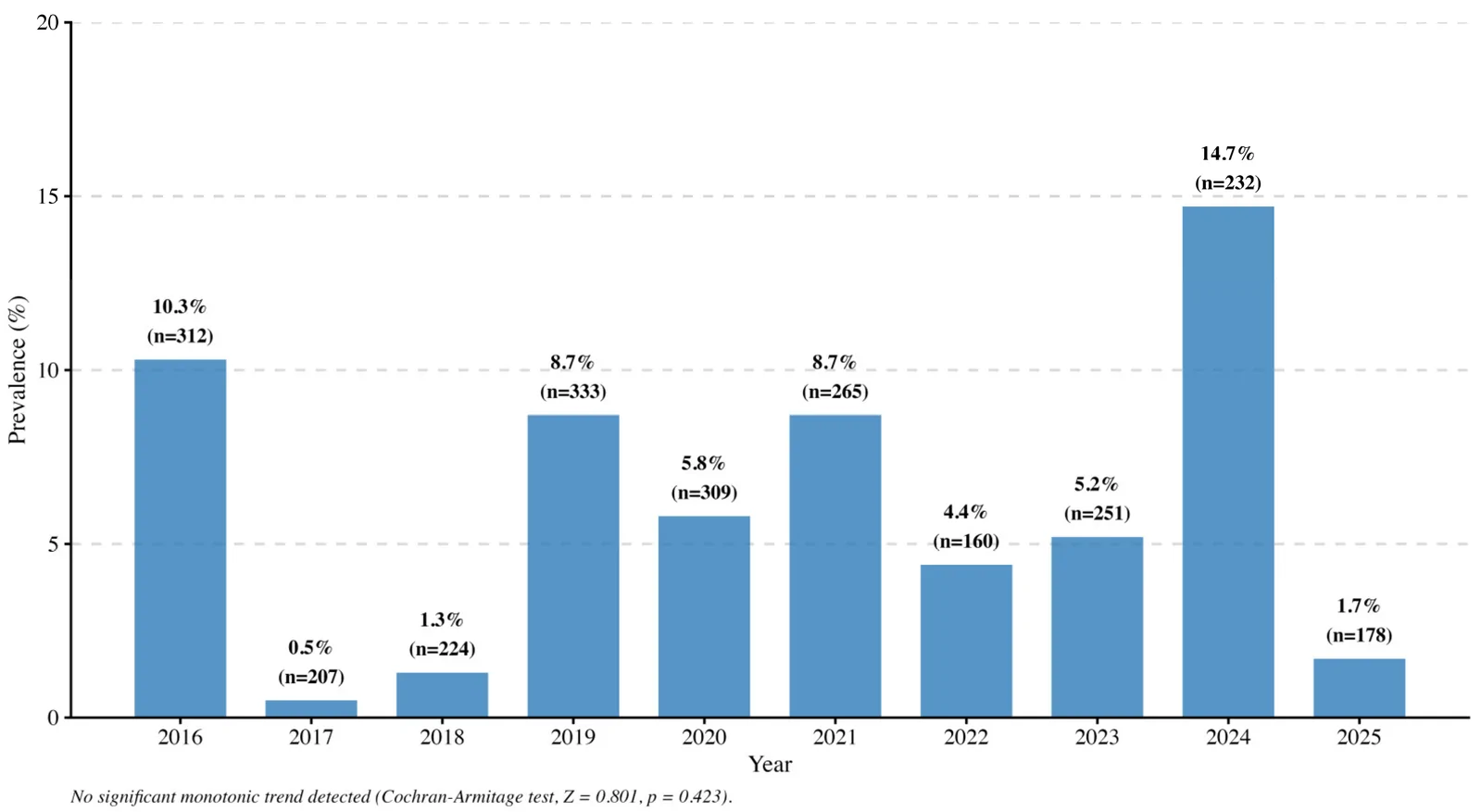
Annual prevalence of *Listeria monocytogenes* in traditional Montenegrin meat products from 2016 to 2025 (*n* = 2471). Values above bars indicate prevalence (%) and total number of samples tested per year. No statistically significant monotonic trend was detected across the surveillance period (Cochran–Armitage test, Z = 0.801, *p* = 0.423).

Over the ten-year period, *L. monocytogenes* was detected in 163 out of 2471 samples, corresponding to an overall prevalence of 6.6%. Annual prevalence estimates ranged from 0.5% (2017; 1/207) to 14.7% (2024; 34/232), demonstrating considerable inter-annual variability (Figure 1). Higher annual estimates were observed in 2016 (10.3%; 32/312), 2019 (8.7%; 29/333), 2021 (8.7%; 23/265), and 2024 (14.7%; 34/232), whereas 2017 (0.5%; 1/207), 2018 (1.3%; 3/224), and 2025 (1.7%; 3/178) recorded the lowest values. The Cochran–Armitage trend test revealed no statistically significant monotonic trend across the surveillance period (Z = 0.801, *p* = 0.423), indicating that annual prevalence varied considerably from year to year without evidence of a systematic directional change in either direction.

### 3.3. Prevalence by Product Category

**Figure 2:**
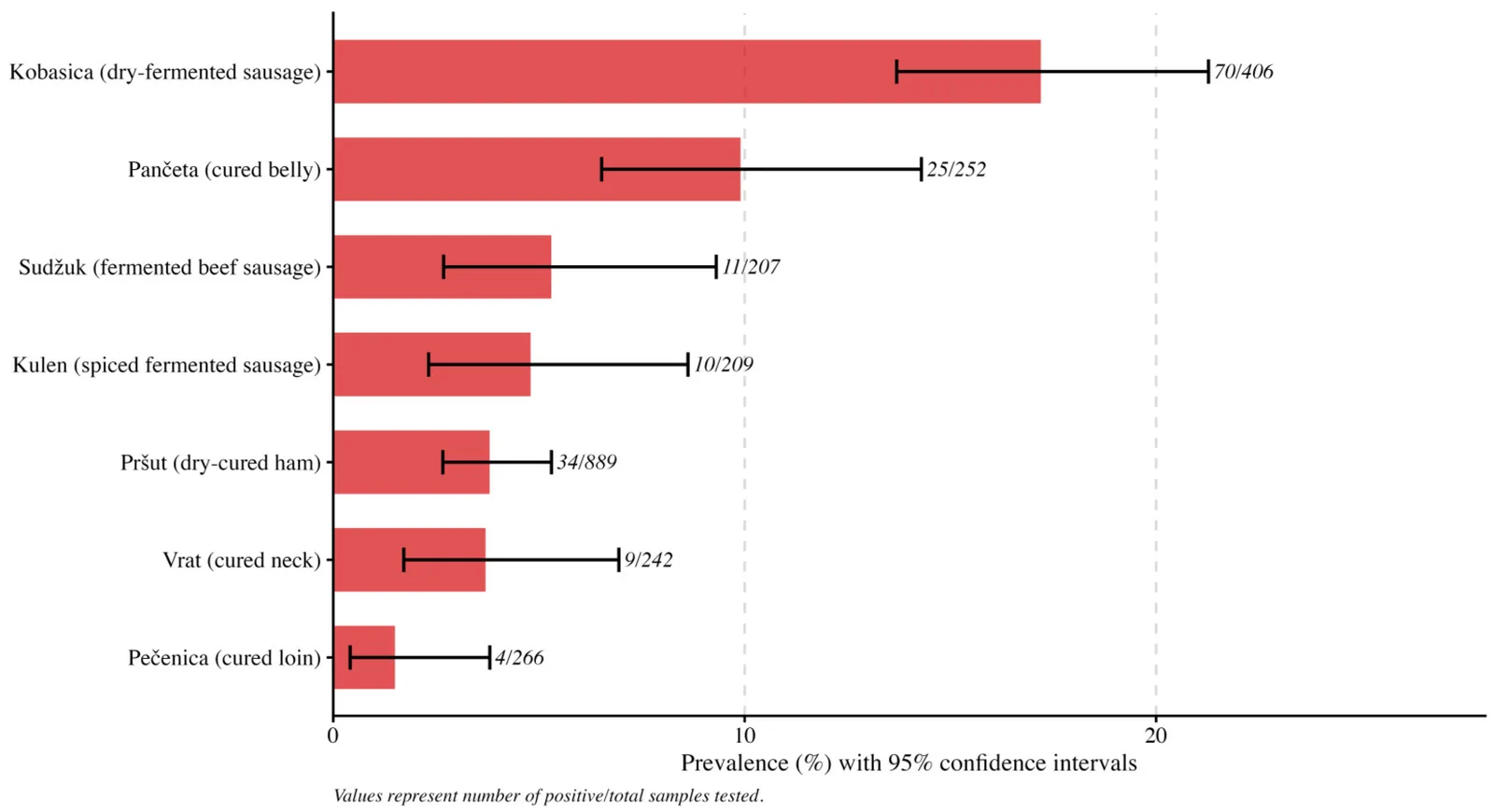
*Listeria monocytogenes* prevalence by product category in traditional Montenegrin meat products, 2016–2025, (*n* = 2471). Error bars represent 95% confidence intervals calculated using the exact binomial (Clopper–Pearson) method. Values to the right of error bars indicate the number of positive samples relative to total samples tested. Categories are ordered by decreasing prevalence.

Statistically significant differences in *L. monocytogenes* prevalence were observed across product categories (χ^2^ = 106.39, df = 6, *p* < 0.001; Figure 2). Dry-fermented sausages (kobasica) recorded the highest prevalence (17.2%; 70/406; 95% CI: 13.6–21.3%), followed by cured belly (pančeta; 9.9%; 25/252; 95% CI: 6.5–14.2%). Fermented beef sausage (sudžuk) showed a prevalence of 5.3% (11/207; 95% CI: 2.7–9.3%), followed by spiced fermented sausage (kulen; 4.8%; 10/209; 95% CI: 2.3–8.6%). Prosciutto (pršut; 3.8%; 34/889; 95% CI: 2.7–5.3%) and cured neck (vrat; 3.7%; 9/242; 95% CI: 1.7–6.9%) exhibited similar and significantly lower prevalence compared to kobasica (*p* < 0.001 for both comparisons after Bonferroni correction). Cured loin (pečenica) recorded the lowest prevalence among all categories (1.5%; 4/266; 95% CI: 0.4–3.8%).

Post hoc pairwise comparisons confirmed that kobasica differed significantly from all other categories (*p* ≤ 0.001 after Bonferroni correction). Pančeta differed significantly from pečenica (*p* = 0.001) and pršut (*p* = 0.005). No significant differences were detected among the remaining category pairs (Table 1).

### 3.4. Prevalence by Individual Product

At the level of individual product varieties, Njeguška kobasica vacuum-packed (Njeguška kobasica vakum pak.) recorded the highest prevalence among all products with a minimum of five samples tested (47.6%; 10/21), followed by Njeguška kobasica (30.1%; 50/166), and Njeguška suva pančeta (22.2%; 10/45) (Table 2). Notably, Crnogorska domaća kobasica (12.5%; 5/40), Crnogorska pančeta (11.6%; 8/69), and Njeguška pančeta (11.4%; 5/44) also exceeded the 10% prevalence threshold. Goveđi kulen (9.4%; 10/106) and Goveđi sudžuk (5.9%; 11/185) represented the highest-risk products within their respective categories. In contrast, several product varieties with substantial sample sizes recorded zero detections, including Kulen (0/89), Kulen–fermentisana suva kobasica (0/60), Njeguška suva pečenica (0/41), and Njeguški pršut kocka (0/25).

### 3.5. Temporal Patterns by Product Category

**Figure 3:**
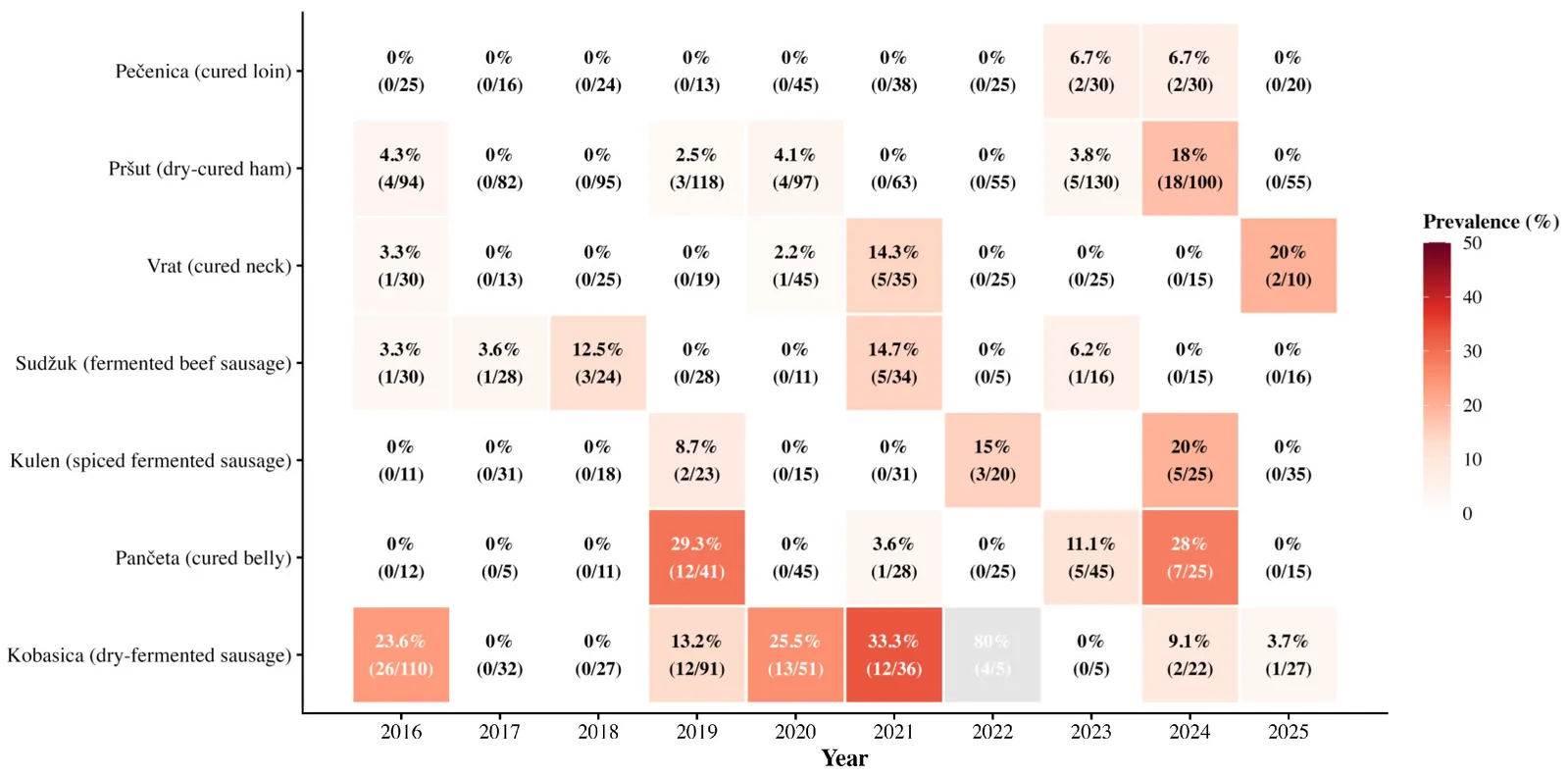
Heatmap of *Listeria monocytogenes* prevalence by product category and year (2016–2025). Cell values indicate prevalence (%) and the number of positive/total samples tested. Cells labelled “*n* < 5” contained fewer than five samples and are shown in grey; such cells (e.g., kobasica 2022, 4/5) should be interpreted with caution due to limited statistical power. Colour intensity reflects prevalence magnitude, ranging from white (0%) to dark red (≥50%).

The heatmap analysis (Figure 3) revealed distinct temporal clustering of contamination events across product categories. Kobasica exhibited the most persistent contamination pattern, with elevated prevalence recorded in 2016 (23.6%; 26/110), 2019 (13.2%; 12/91), 2020 (25.5%; 13/51), and 2021 (33.3%; 12/36), with a notable outlier in 2022 (80.0%; 4/5), though the latter is based on a very small sample size and should be interpreted with caution. Pančeta showed pronounced peaks in 2019 (29.3%; 12/41) and 2024 (28.0%; 7/25). Pršut recorded its highest contamination rate in 2024 (18.0%; 18/100), coinciding with the overall annual peak observed that year. Sudžuk demonstrated elevated prevalence in 2018 (12.5%; 3/24) and 2021 (14.7%; 5/34), with negligible contamination in intervening years. Kulen showed contamination primarily in 2022 (15.0%; 3/20) and 2024 (20.0%; 5/25), while pečenica and vrat remained largely free of contamination across most years, with sporadic detections in 2023–2024 and 2021/2025, respectively.

### 3.6. Prevalence by Inspection Type

Prevalence estimates varied markedly according to the type of inspection (Table 3). The highest prevalence was recorded among samples submitted through official veterinary inspection of production facilities (12.9%; 11/85), followed by on-request production inspection (7.8%; 48/617) and contractual production monitoring (7.6%; 85/1116). Official monitoring of retail products yielded a markedly lower prevalence of 0.4% (1/234), consistent with the expectation that end-of-shelf-life competitive microbiota pressure reduces pathogen viability. All imported samples tested negative regardless of inspection category (0/85), suggesting either effective border control or selective sampling of lower-risk product types.

### 3.7. Quantitative Findings

Among all traditional meat product categories examined, a single quantitative positive result was identified: Njeguška kobasica sampled in 2019 during official retail monitoring yielded a count of <10 CFU/g, a value below the regulatory limit of 100 CFU/g established under Commission Regulation (EC) No 2073/2005 for ready-to-eat foods unable to support the growth of *L. monocytogenes*. No quantitative results exceeding the legal threshold were detected within the traditional product subset, distinguishing this category from the broader dataset in which counts as high as 4.8 × 10^6^ CFU/g were recorded in non-traditional RTE products.

## 4. Discussion

### 4.1. Overall Prevalence in Context

The overall *L. monocytogenes* prevalence of 6.6% observed across traditional Montenegrin meat products in this study is markedly higher than the average prevalence reported for ready-to-eat (RTE) meat products in the European Union, where official sampling at manufacturing and distribution stages generally ranges from 0% to 2.8%, with fermented sausages representing a notable exception at approximately 14.8% positivity at distribution level [3]. The prevalence detected in our study is consistent with previous findings demonstrating that traditional and artisanal dry-fermented and dry-cured meat products, characterised by reliance on autochthonous house microbiota and absence of standardised starter cultures, represent a higher contamination risk than industrially produced counterparts [17,18]. Comparable prevalence values have been reported in studies of Turkish sucuk (11.6% positivity across 300 retail samples [16]), Italian dry-fermented sausages from small-scale Sardinian producers (31.5% in food samples and 68.5% in environmental samples across four processing plants [17]), and French dried sausage processing plants, where surface swab positivity ranged from 15% before working hours to 47% during active processing across 13 facilities [18], reinforcing the conclusion that the artisanal manufacturing model carries inherent microbiological risk that requires sustained surveillance. A recent systematic review of quantitative risk assessment models for *L. monocytogenes* in meat and meat products has similarly identified fermented sausages and dry-cured pork as processing categories in which risk-based intervention strategies remain particularly warranted [27].

The Montenegrin context is particularly relevant given prior molecular characterisation work in the country. Zuber et al. (2019) documented persistent contamination of a small-scale Montenegrin meat processing facility producing traditional products over a four-year period (2011–2014), identifying four major sequence types (ST515, ST8, ST21, ST121) and indicating that contamination most likely originated from incoming raw materials [23]. More recently, Daza Prieto et al. (2024) applied whole-genome sequencing to 160 *L. monocytogenes* isolates recovered from a broader sampling of 22,593 food-chain sources in Montenegro during 2014–2022, revealing a high genetic diversity (21 clonal complexes, 73 cgMLST types) with ST8, ST9, ST121 and ST155 as the most prevalent sequence types [22]. Similar clonal patterns have been documented in the neighbouring Republic of Kosovo, where MLST analysis of *L. monocytogenes* isolates from routine food surveillance identified overlapping sequence types across ready-to-eat products and processing environments [28], reinforcing the interpretation that a shared regional pool of persistent clones circulates across the Western Balkans food chain. The persistence of these clones across multiple years and facilities, combined with the absence of any significant monotonic trend in our ten-year prevalence data (*p* = 0.423), strongly suggests that *L. monocytogenes* has established stable ecological niches within the Montenegrin traditional meat production environment.

### 4.2. Product Category-Specific Risk

The marked differences in prevalence observed across product categories (χ^2^ = 106.39, *p* < 0.001) reflect the distinct physicochemical and processing characteristics of each product type. Dry-fermented sausages (kobasica; 17.2%) recorded the highest prevalence, significantly exceeding all other categories. This finding is mechanistically consistent with the production technology: dry-fermented sausages rely on spontaneous fermentation by autochthonous lactic acid bacteria without thermal processing, and their relatively high water activity during the early fermentation phase (*a*_w_ > 0.92) provides a permissive environment for *L. monocytogenes* survival and even growth before sufficient acidification and drying occur [14,29]. Janković et al. (2017) demonstrated that *L. monocytogenes* detected at the beginning of the production cycle of Petrovská klobása sausage in Serbia disappeared by day 30 of processing, but that pathogen decline was substantially slower under traditional household drying conditions compared with controlled industrial drying [28], a finding directly relevant to the small-scale, artisanal nature of Montenegrin kobasica production.

The lower prevalence observed for pršut (3.8%) is similarly explainable by the product’s physicochemical profile. Serra-Castelló et al. (2020) demonstrated that the extended dry-curing process, which progressively reduces water activity to values below 0.92, exerts a listericidal rather than merely listeriostatic effect on *L. monocytogenes*, with measurable inactivation rates dependent on final *a*_w_ and storage temperature [30]. The contamination that does occur in pršut is therefore more likely attributable to post-processing cross-contamination during deboning, slicing, or packaging, as documented in Spanish dry-cured ham facilities by Alía et al. (2020) [8] and in the Parma ham production chain by Morganti et al. (2016) [7]. This interpretation is supported in our dataset by the exceptionally high prevalence observed in Njeguška kobasica vacuum-packed (Njeguška kobasica vakum pak.; 47.6%) compared with the bulk product (30.1%), which is consistent with—though does not prove—the possibility that the slicing and packaging stages represent critical control points where post-processing contamination may occur.

Sudžuk and kulen (4.8%) share the fermented sausage technology with kobasica but differ in raw material composition (beef versus pork) and spice formulation. The lower prevalence in these categories cannot be attributed to any single mechanism on the basis of the present data. Differences in raw material microbiota, starter dynamics, and processing duration may all contribute. Spices such as garlic and red pepper are traditionally incorporated into these products, and their potential antimicrobial activity against *L. monocytogenes* has been reported in vitro [31]; however, spices can also act as vectors of microbial contamination during cultivation, drying, and storage [32,33], and the net effect in a specific fermented meat matrix cannot be inferred from prevalence data alone. The lowest prevalence values were recorded in pečenica (1.5%), vrat (3.7%), and pršut (3.8%), all of which are whole-muscle products subject to extended dry-curing without comminution, a processing approach that limits opportunities for cross-contamination and provides sustained antimicrobial hurdle conditions.

### 4.3. Product-Level Findings and High-Risk Varieties

At the level of individual product varieties, Njeguška kobasica and its vacuum-packed variant emerged as the products of greatest concern, with prevalence values of 30.1% and 47.6%, respectively. These findings warrant particular attention given the cultural and economic significance of Njeguška kobasica as a flagship traditional product of Montenegro. The higher prevalence in the vacuum-packed format compared with the bulk product could be consistent with the possibility that the packaging operation is performed under conditions permitting environmental contamination. This remains a hypothesis: no significance test was performed between the two formats, sample sizes are unequal (*n* = 21 vs. *n* = 166), and product-level identifiers were not available. The hypothesis is nevertheless plausible in light of the WGS findings of Zuber et al. (2019), who identified persistent ST121 strains in the processing environment of a Montenegrin facility producing traditional meat products [23], and warrants targeted investigation of slicing and packaging environments. ST121 is internationally recognised as a persistent, biofilm-forming clone strongly associated with food processing environments [22], and its presence in the Montenegrin food chain represents a structural challenge that cannot be resolved by end-product testing alone.

Conversely, several product varieties recorded zero detections despite substantial sample sizes, most notably Kulen (0/89), Kulen–fermentisana suva kobasica (0/60), and Njeguška suva pečenica (0/41). The contrast between Goveđi kulen (9.4%) and Kulen (0%) is particularly informative: although both are spiced fermented sausages, Goveđi kulen is produced from beef while traditional pork-based Kulen may benefit from differences in raw material microbiota, processing duration, or producer-specific hygiene practices. These distinctions reinforce the conclusion that risk profiles cannot be inferred from product category alone but require product-specific risk-based monitoring.

### 4.4. Temporal Patterns and Absence of a Downward Trend

The absence of a statistically significant monotonic trend over the ten-year surveillance period (Cochran–Armitage Z = 0.801, *p* = 0.423) is one of the most policy-relevant findings of this study. Annual prevalence estimates varied considerably from year to year, but no systematic directional change was observed in either direction, despite Montenegro’s progressive alignment with EU food safety legislation, the establishment of the National Reference Laboratory framework, and increased awareness among food business operators. This pattern mirrors the broader European trajectory: the EFSA-ECDC One Health 2024 Zoonoses Report [4] documented that confirmed invasive human listeriosis cases have shown a significant increasing trend over the last five years, despite intensified regulatory and surveillance efforts across Member States [3]. Recent global reviews consolidating surveillance and recall data from Europe, North America, and Australia have similarly concluded that *L. monocytogenes* remains a persistent public-health concern in RTE food supply chains despite established regulatory frameworks and intervention strategies [34].

The observed inter-annual variability, higher annual estimates in 2016 (10.3%), 2019 (8.7%), 2021 (8.7%), and 2024 (14.7%), likely reflects episodic contamination events at the level of individual producers rather than systemic shifts in the food chain. The 2024 peak, driven predominantly by elevated prevalence in pršut (18.0%) and pančeta (28.0%), is particularly noteworthy and may indicate a specific contamination event at one or more production facilities. Such episodic patterns underscore the value of continuous, long-term surveillance and the need to integrate phenotypic prevalence data with molecular subtyping to identify and remediate persistent contamination sources.

### 4.5. Inspection-Type Effects and the Case for Environmental Monitoring

The substantially higher prevalence observed in production-stage samples (7.6–12.9%) compared with retail-stage monitoring samples (0.4%) carries important regulatory implications. End-of-shelf-life retail samples, by virtue of their physicochemical maturation (reduced *a*_w_, lower pH, established competitive microbiota), may yield lower detection rates not because contamination has been controlled but because the pathogen has been progressively inactivated during product ageing [29]. This phenomenon, while protective from a final-product safety perspective, masks the underlying contamination pressure at the production stage and provides false reassurance regarding the hygienic status of facilities. Our findings argue for greater emphasis on production-stage monitoring as a complement to end-product testing, an orientation reinforced by Commission Regulation (EU) 2024/2895, which extends the food safety criterion “*L. monocytogenes* not detected in 25 g” across the entire shelf-life of RTE foods able to support pathogen growth, rather than only at the point at which products leave the producer’s control [5].

The complete absence of positive results in imported products (0/85) requires cautious interpretation. While this may reflect effective border control and the lower-risk product types typically subjected to import inspection, the limited sample size precludes firm conclusions about the comparative safety of imported versus domestically produced traditional meat products.

### 4.6. Public Health Significance

Although Montenegro is not currently included in the EFSA-ECDC zoonoses surveillance system and listeriosis case data for the country are not systematically reported, the regional context is informative. Croatia and Romania, the nearest reporting EU Member States, register the lowest listeriosis notification rates in the EU (≤0.20 per 100,000 population) [3], which may reflect either genuinely low incidence or under-diagnosis and under-reporting. Given the high prevalence of *L. monocytogenes* documented in this study and the elevated consumption of traditional dry-cured and fermented meat products among the Montenegrin population, the possibility that listeriosis is under-recognised in the Western Balkans warrants further epidemiological investigation. The recent updates to Commission Regulation (EC) No 2073/2005 [11], which will require demonstration of growth-limiting characteristics throughout shelf-life rather than only at the end of producer control (Commission Regulation (EU) 2024/2895) [5], will impose additional regulatory pressure on producers of traditional RTE meats, a challenge that will require coordinated technical support, particularly for small-scale artisanal producers who form the backbone of the Montenegrin traditional meat sector.

### 4.7. Limitations

Several limitations should be acknowledged. First, only one quantitative result was available within the traditional product subset, precluding meaningful exposure assessment based on enumeration data. Second, the dataset does not include molecular characterisation of isolates, limiting the ability to distinguish between recurrent contamination from persistent strains and episodic contamination from independent sources. Third, and most importantly for the interpretation of temporal patterns, the distribution of samples across inspection types was heterogeneous both between years and within product categories. The overall dataset combines samples originating from contractual monitoring, on-request veterinary inspection, official routine monitoring, and import control, with varying proportions of each in different years. Because these inspection modes differ in their underlying sampling logic (routine random-like coverage versus targeted risk-based sampling), the composition of the sample set in a given year can substantially influence the annual prevalence estimate independently of any true change in contamination pressure. The apparent peaks and troughs observed in Figure 1 and Figure 3, including the pronounced 2024 estimate, could therefore reflect year-to-year shifts in sampling composition rather than genuine changes in food-chain contamination. Individual high-prevalence cells in the heatmap (e.g., kobasica 2022, 4/5) can be driven by one or two producers or even a single production batch and should not be extrapolated to the whole category or year. Fourth, sampling intensity varied across years and producers, and the sample distribution by producer is not uniformly representative of the Montenegrin traditional meat sector as a whole. Fifth, the operational definition of “traditional” used here, while pragmatic, may not fully align with regulatory or geographical indication frameworks. Future work should integrate prevalence data with whole-genome sequencing, environmental sampling, and quantitative microbial risk assessment to provide a comprehensive picture of *L. monocytogenes* dynamics in traditional Montenegrin meat products.

## 5. Conclusions

This study presents the first systematic, decade-long surveillance assessment of *Listeria monocytogenes* in traditional Montenegrin dry-cured and fermented meat products, based on 2471 samples across 58 product varieties tested between 2016 and 2025. The overall prevalence of 6.6% substantially exceeds typical European Union values for officially sampled ready-to-eat meat products and is consistent with the contamination risk previously documented for artisanal and small-scale fermented meat production.

Product category emerged as a strong predictor of contamination risk, with dry-fermented sausages (kobasica; 17.2%) and cured belly (pančeta; 9.9%) recording prevalence values significantly higher than whole-muscle dry-cured products (pršut, pečenica, vrat; 1.5–3.8%). At the individual product level, Njeguška kobasica and its vacuum-packed variant emerged as the highest-risk varieties, with prevalence values of 30.1% and 47.6% respectively, identifying a specific point of intervention within the Montenegrin traditional meat sector. The substantially higher prevalence in production-stage samples compared with retail samples suggests that current end-product testing underestimates true contamination pressure at the source and supports a regulatory shift toward systematic environmental monitoring in production facilities.

Critically, no statistically significant monotonic trend was detected over the ten-year period (Cochran–Armitage, Z = 0.801, *p* = 0.423), indicating that annual prevalence varied considerably from year to year without any systematic directional change. This finding is consistent with the broader European trajectory, where confirmed invasive listeriosis cases have shown a significant increasing trend over the last five years [4].

These results have direct implications for food safety policy in Montenegro and the wider Western Balkans. Effective control of *L. monocytogenes* in traditional meat products will require a coordinated, multi-pronged strategy combining (i) systematic environmental monitoring of production facilities, particularly at critical control points such as slicing and packaging operations; (ii) integration of whole-genome sequencing into surveillance programmes to identify and remediate persistent strains; (iii) targeted technical support for small-scale artisanal producers, who form the backbone of the traditional meat sector and face disproportionate compliance burdens under the strengthened requirements of Commission Regulation (EU) 2024/2895; and (iv) establishment of a national listeriosis surveillance system to enable epidemiological linkage between food contamination data and human disease outcomes. The high genetic diversity and persistence of *L. monocytogenes* clones previously documented in the Montenegrin food chain, combined with the prevalence demonstrated in this study, indicate that traditional Montenegrin meat products warrant prioritisation within the national food safety agenda and serve as a useful sentinel category for evaluating the effectiveness of future interventions.

## Figures and Tables

**Table 1:** Post hoc pairwise comparisons of *Listeria monocytogenes* prevalence between traditional meat product categories (Pearson chi-square with Bonferroni correction).

Category 1	Category 2	χ^2^ *p*-Value	Bonferroni-Adjusted *p*-Value	Significant
Kobasica (dry-fermented sausage)	Pršut (dry-cured ham)	4.23 × 10^−16^	8.89 × 10^−15^	Yes
Kobasica (dry-fermented sausage)	Pečenica (cured loin)	4.18 × 10^−10^	8.77 × 10^−9^	Yes
Kobasica (dry-fermented sausage)	Vrat (cured neck)	6.87 × 10^−7^	1.44 × 10^−5^	Yes
Kobasica (dry-fermented sausage)	Kulen (spiced fermented sausage)	2.41 × 10^−5^	5.06 × 10^−4^	Yes
Kobasica (dry-fermented sausage)	Sudžuk (fermented beef sausage)	6.39 × 10^−5^	0.001	Yes
Pančeta (cured belly)	Pečenica (cured loin)	7.08 × 10^−5^	0.001	Yes
Pančeta (cured belly)	Pršut (dry-cured ham)	2.19 × 10^−4^	0.005	Yes
Pančeta (cured belly)	Vrat (cured neck)	1.10 × 10^−2^	0.230	No
Kobasica (dry-fermented sausage)	Pančeta (cured belly)	1.30 × 10^−2^	0.273	No
Pečenica (cured loin)	Sudžuk (fermented beef sausage)	3.74 × 10^−2^	0.785	No
Kulen (spiced fermented sausage)	Pančeta (cured belly)	5.80 × 10^−2^	1.000	No
Kulen (spiced fermented sausage)	Pečenica (cured loin)	6.79 × 10^−2^	1.000	No
Kulen (spiced fermented sausage)	Pršut (dry-cured ham)	6.59 × 10^−1^	1.000	No
Kulen (spiced fermented sausage)	Sudžuk (fermented beef sausage)	9.82 × 10^−1^	1.000	No
Kulen (spiced fermented sausage)	Vrat (cured neck)	7.44 × 10^−1^	1.000	No
Pančeta (cured belly)	Sudžuk (fermented beef sausage)	9.85 × 10^−2^	1.000	No
Pečenica (cured loin)	Pršut (dry-cured ham)	9.58 × 10^−2^	1.000	No
Pečenica (cured loin)	Vrat (cured neck)	1.94 × 10^−1^	1.000	No
Pršut (dry-cured ham)	Sudžuk (fermented beef sausage)	4.36 × 10^−1^	1.000	No
Pršut (dry-cured ham)	Vrat (cured neck)	1.00 × 10^0^	1.000	No
Sudžuk (fermented beef sausage)	Vrat (cured neck)	5.57 × 10^−1^	1.000	No

**Table 2:** *Listeria monocytogenes* prevalence by individual traditional meat product variety (products with ≥5 samples tested). Products are ordered by decreasing prevalence; products with zero detections are listed alphabetically at the end of the table.

Product	Category	Total Samples (*n*)	Positive Samples (*n*)	Prevalence (%)
Njeguška kobasica vakum pak.	Kobasica	21	10	47.6
Njeguška kobasica	Kobasica	166	50	30.1
Njeguška suva panceta	Pančeta	45	10	22.2
Crnogorska domaća kobasica	Kobasica	40	5	12.5
Crnogorska panceta	Pančeta	69	8	11.6
Njeguška panceta	Pančeta	44	5	11.4
Goveđi kulen	Kulen	106	10	9.4
Crnogorski pršut	Pršut	185	14	7.6
Njeguški vrat	Vrat	94	6	6.4
Goveđi sudžuk	Sudžuk	185	11	5.9
Njeguška domaća kobasica	Kobasica	20	1	5.0
Njeguški pršut	Pršut	224	10	4.5
Crnogorska kobasica	Kobasica	23	1	4.3
Crnogorski vrat	Vrat	47	2	4.3
Podlovćenska panceta	Pančeta	55	2	3.6
Pršut crnogorski	Pršut	193	7	3.6
Crnogorska pečenica	Pečenica	65	2	3.1
Njeguška pečenica	Pečenica	107	2	1.9
Podlovćenska pršuta	Pršut	54	1	1.9
Podlovćenski vrat	Vrat	53	1	1.9
Goveđa bjelopoljska kobasica	Kobasica	60	1	1.7
Njeguška pršuta	Pršut	131	2	1.5
Ceklićka pečenica	Pečenica	7	0	0.0
Crnogorska suva panceta list	Pančeta	11	0	0.0
Crnogorska suva pečenica list	Pečenica	6	0	0.0
Crnogorska suva pršuta list	Pršut	12	0	0.0
Crnogorski suvi vrat u listu	Vrat	10	0	0.0
Juneći kulen	Kulen	14	0	0.0
Kulen	Kulen	89	0	0.0
Kulen–fermentisana suva kobasica	Kobasica	60	0	0.0
Lovćenska kobasica	Kobasica	9	0	0.0
Lovćenska pečenica	Pečenica	5	0	0.0
Lovćenski vrat	Vrat	5	0	0.0
Njeguška pančeta komad vakum pak.	Pančeta	13	0	0.0
Njeguška suva pečenica	Pečenica	41	0	0.0
Njeguški but	Pršut	5	0	0.0
Njeguški pršut kocka	Pršut	25	0	0.0
Njeguški suvi pršut	Pršut	5	0	0.0
Njeguški suvi vrat	Vrat	20	0	0.0
Podlovćenska pečenica	Pečenica	30	0	0.0
Podlovćenska pršuta, slajs vak	Pršut	7	0	0.0
Podlovćenski pršut	Pršut	20	0	0.0
Sudžuk	Sudžuk	22	0	0.0
Suvi njeguški pršut BK	Pršut	5	0	0.0
Suvi njeguški pršut rezani	Pršut	5	0	0.0
Vrat podlovćenski	Vrat	5	0	0.0
Ćeklićka panceta	Pančeta	11	0	0.0
Ćeklićka pečenica	Pečenica	5	0	0.0
Ćeklićka pršuta	Pršut	18	0	0.0
Ćeklićki vrat	Vrat	5	0	0.0

**Table 3:** *Listeria monocytogenes* prevalence in traditional Montenegrin meat products stratified by type of official inspection programme (2016–2025). Inspection categories reflect the administrative framework of the Montenegrin national food control system.

Inspection Type	Total Samples (*n*)	Positive Samples (*n*)	Prevalence (%)
Contractual monitoring—production stage	1116	85	7.6
On-request testing—production stage	617	48	7.8
Official veterinary monitoring—retail stage	234	1	0.4
Official veterinary monitoring—production stage	105	10	9.5
Contractual monitoring—unspecified stage	95	1	1.1
Veterinary inspector-requested—production stage	85	11	12.9
On-request testing—unspecified stage	83	5	6.0
Import inspection—veterinary	50	0	0.0
Veterinary inspector-requested—unspecified stage	21	2	9.5
Import inspection—third-party laboratories	20	0	0.0
Import inspection—general	15	0	0.0
Official veterinary monitoring—unspecified stage	10	0	0.0
On-request testing—retail stage	10	0	0.0
Veterinary ambulance-requested testing	7	0	0.0
Contractual monitoring—retail stage	3	0	0.0

## Data Availability

All data underlying the reported analyses are presented in the manuscript itself (Table 2 and Table 3, Figure 1, Figure 2 and Figure 3). The complete anonymised dataset, including individual record-level testing results, is available from the corresponding author upon reasonable request, subject to institutional approval by the Diagnostic Veterinary Laboratory of Montenegro.

## Abbreviations

The following abbreviations are used in this manuscript:

CFU	Colony-forming unit
CC	Clonal complex
CI	Confidence interval
EC	European Commission
ECDC	European Centre for Disease Prevention and Control
EFSA	European Food Safety Authority
EU	European Union
GI	Geographical indication
ISO	International Organization for Standardization
RTE	Ready-to-eat
ST	Sequence type
WGS	Whole-genome sequencing

## References

[1] Radoshevich L., Cossart P. (2018). *Listeria monocytogenes*: Towards a Complete Picture of Its Physiology and Pathogenesis. Nat. Rev. Microbiol..

[2] Charlier C., Perrodeau É., Leclercq A., Cazenave B., Pilmis B., Henry B., Lopes A., Maury M.M., Moura A., Goffinet F. (2017). Clinical Features and Prognostic Factors of Listeriosis: The MONALISA National Prospective Cohort Study. Lancet Infect. Dis..

[3] European Food Safety Authority (EFSA), European Centre for Disease Prevention and Control (ECDC) (2024). The European Union One Health 2023 Zoonoses Report. EFSA J..

[4] European Food Safety Authority (EFSA), European Centre for Disease Prevention and Control (ECDC) (2025). The European Union One Health 2024 Zoonoses Report. EFSA J..

[5] European Commission (2024). Commission Regulation (EU) 2024/2895 of 19 November 2024 Amending Regulation (EC) No 2073/2005 as Regards Listeria monocytogenes.

[6] Thevenot D., Dernburg A., Vernozy-Rozand C. (2006). An Updated Review of *Listeria monocytogenes* in the Pork Meat Industry and Its Products. J. Appl. Microbiol..

[7] Morganti M., Scaltriti E., Cozzolino P., Bolzoni L., Casadei G., Pierantoni M., Foni E., Pongolini S. (2016). Processing-Dependent and Clonal Contamination Patterns of *Listeria monocytogenes* in the Cured Ham Food Chain Revealed by Genetic Analysis. Appl. Environ. Microbiol..

[8] Alía A., Andrade M.J., Rodríguez A., Martín I., Pérez-Baltar A., Medina M., Córdoba J.J. (2020). Prevalence and Characterization of *Listeria monocytogenes* in Deboning and Slicing Areas of Spanish Dry-Cured Ham Processing. LWT.

[9] NicAogáin K., O’Byrne C.P. (2016). The Role of Stress and Stress Adaptations in Determining the Fate of the Bacterial Pathogen *Listeria monocytogenes* in the Food Chain. Front. Microbiol..

[10] Gandhi M., Chikindas M.L. (2007). *Listeria*: A Foodborne Pathogen That Knows How to Survive. Int. J. Food Microbiol..

[11] European Commission (2005). Commission Regulation (EC) No 2073/2005 of 15 November 2005 on Microbiological Criteria for Foodstuffs. Off. J. Eur. Union.

[12] Vermeulen A., Gysemans K.P.M., Bernaerts K., Geeraerd A.H., Van Impe J.F., Debevere J., Devlieghere F. (2007). Influence of PH, Water Activity and Acetic Acid Concentration on *Listeria monocytogenes* at 7 °C: Data Collection for the Development of a Growth/No Growth Model. Int. J. Food Microbiol..

[13] Tienungoon S., Ratkowsky D.A., McMeekin T.A., Ross T. (2000). Growth Limits of *Listeria monocytogenes* as a Function of Temperature, PH, NaCl, and Lactic Acid. Appl. Environ. Microbiol..

[14] Talon R., Leroy S., Lebert I. (2007). Microbial Ecosystems of Traditional Fermented Meat Products: The Importance of Indigenous Starters. Meat Sci..

[15] Aymerich T., Picouet P.A., Monfort J.M. (2008). Decontamination Technologies for Meat Products. Meat Sci..

[16] Colak H., Hampikyan H., Ulusoy B., Bingol E.B. (2007). Presence of *Listeria monocytogenes* in Turkish Style Fermented Sausage (Sucuk). Food Control.

[17] Mureddu A., Mazza R., Fois F., Meloni D., Bacciu R., Piras F., Mazzette R. (2014). *Listeria monocytogenes* Persistence in Ready-to-Eat Sausages and in Processing Plants. Ital. J. Food Saf..

[18] Thévenot D., Delignette-Muller M.L., Christieans S., Vernozy-Rozand C. (2005). Prevalence of *Listeria monocytogenes* in 13 Dried Sausage Processing Plants and Their Products. Int. J. Food Microbiol..

[19] Jashari B., Capitaine K., Bisha B., Stessl B., Blagoevska K., Cana A., Jankuloski D., Félix B. (2024). Molecular Characterization of *Listeria monocytogenes* in the Food Chain of the Republic of Kosovo from 2016 to 2022. Foods.

[20] Simunovic S., Đorđević V., Barba F.J., Lorenzo J.M., Rašeta M., Janković S., Tomasevic I. (2021). Characterisation of Changes in Physicochemical, Textural and Microbiological Properties of Njeguška Sausage during Ripening. J. Food Sci. Technol..

[21] Pleadin J., Lešić T., Vujačić V., Milićević D., Buneta A., Šušnić S., Lukanić I., Krešić G. (2021). Comparison of Chemical Composition and Fatty Acid Profile of Traditional Meat Products from Croatia and Montenegro. J. Food Qual..

[22] Daza Prieto B., Pietzka A., Martinovic A., Ruppitsch W., Zuber Bogdanovic I. (2024). Surveillance and Genetic Characterization of *Listeria monocytogenes* in the Food Chain in Montenegro during the Period 2014–2022. Front. Microbiol..

[23] Zuber I., Lakicevic B., Pietzka A., Milanov D., Djordjevic V., Karabasil N., Teodorovic V., Ruppitsch W., Dimitrijevic M. (2019). Molecular Characterization of *Listeria monocytogenes* Isolates from a Small-Scale Meat Processor in Montenegro, 2011–2014. Food Microbiol..

[24] (2022). Microbiology of the Food Chain—Horizontal Method for the Detection and Enumeration of *Listeria monocytogenes* and of *Listeria* spp.—Part 1: Detection Method.

[25] (2017). Microbiology of the Food Chain—Horizontal Method for the Detection and Enumeration of *Listeria monocytogenes* and of *Listeria* spp.—Part 2: Enumeration Method.

[26] R Core Team (2022). R: A Language and Environment for Statistical Computing.

[27] Gonzales-Barron U., Cadavez V., De Oliveira Mota J., Guillier L., Sanaa M. (2024). A Critical Review of Risk Assessment Models for *Listeria monocytogenes* in Meat and Meat Products. Foods.

[28] Jashari B., Stessl B., Félix B., Cana A., Bisha B., Jankuloski D., Blagoevska K., Kayode A.J. (2024). Multilocus Sequence Typing and Antimicrobial Susceptibility of *Listeria monocytogenes* Isolated from Foods Surveyed in Kosovo. Microorganisms.

[29] Janković V., Mitrović R., Lakićević B., Velebit B., Baltić T. (2017). *Listeria monocytogenes* Presence during Fermentation, Drying and Storage of Petrovská Klobása Sausage. IOP Conf. Ser. Earth Environ. Sci..

[30] Serra-Castelló C., Jofré A., Garriga M., Bover-Cid S. (2020). Modeling and Designing a *Listeria monocytogenes* Control Strategy for Dry-Cured Ham Taking Advantage of Water Activity and Storage Temperature. Meat Sci..

[31] Verluyten J., Leroy F., de Vuyst L. (2004). Effects of Different Spices Used in Production of Fermented Sausages on Growth of and Curvacin A Production by Lactobacillus Curvatus LTH 1174. Appl. Environ. Microbiol..

[32] Gurtler J.B., Keller S.E. (2019). Microbiological Safety of Dried Spices. Annu. Rev. Food Sci. Technol..

[33] Kara R., Gokmen M., Akkaya L., Gok V. (2015). Microbiological Quality and *Salmonella* spp., /*Listeria monocytogenes* of Spices in Turkey. Res. J. Microbiol..

[34] Yangchen J., Sarkar D., Rood L., Vaskoska R., Kocharunchitt C. (2025). *Listeria monocytogenes*: A Continuous Global Threat in Ready-to-Eat (RTE) Foods. Foods.

